# Clinical and Microbiological Profile of Gram-Negative Infections in Critically Ill Diabetic Patients

**DOI:** 10.7759/cureus.65955

**Published:** 2024-08-01

**Authors:** Bhumika Vaishnav, Aniruddh Wadivkar, Ruchitha Pailla, Saish Mondkar

**Affiliations:** 1 General Medicine, Dr. D. Y. Patil Medical College, Hospital & Research Centre, Pune, IND

**Keywords:** gram-negative bacteremia, infections in diabetes, critically ill patients, gram-negative sepsis, diabetes mellitus type 2

## Abstract

Background and aim

Type 2 diabetes mellitus (T2DM) is associated with several infections due to hyperglycemia and impaired immunity. This study aims to analyze the clinical and microbiological profile of critically ill T2DM patients with sepsis due to gram-negative bacteria (GNB).

Materials and methods

A prospective cross-sectional observational study was conducted at Dr. D. Y. Patil Medical College, Hospital & Research Centre, Pune, India, between December 2023 and May 2024, after ethics committee approval. A total of 100 patients (50 T2DM cases and 50 nondiabetic controls), diagnosed with sepsis due to GNB and admitted to the medical ICU, were included in the study. The clinical profile and laboratory investigations of these patients were studied. Cultures were obtained from peripheral/central venous samples, tracheal secretions, and urine samples. Cultures from other specimens, such as ascitic fluid, cerebrospinal fluid, and pus from skin and soft tissue infections, were also obtained. The statistical tests that were applied were two-tailed with a 95% CI, and a p-value of less than 0.05 was considered statistically significant.

Results

The mean age of critically ill T2DM cases was 60.52 ± 12.88 years. Of the 50 T2DM cases, 28 were males and 22 were females. The most common infection in critically ill T2DM patients was bloodstream infection (n = 21), followed by bronchopneumonia (n = 16) and urinary tract infections (n = 10). *Escherichia coli* (n = 15) and *Klebsiella pneumoniae* (n = 15) were the most common gram-negative pathogens isolated. The most common GNB isolated from the blood cultures of critically ill T2DM patients was *Acinetobacter* spp. (n = 6). The death rate was significantly higher in T2DM patients with GNB sepsis as compared to nondiabetic controls.

Conclusion

GNBs like *E. coli*, *K. pneumoniae*, and *Acinetobacter* spp. are commonly found in critically ill T2DM patients with sepsis. Bloodstream infection was the most common site of infection in critically ill T2DM cases. *Acinetobacter* spp. was the most common isolate found in the blood cultures of critically ill T2DM patients. It is important to identify the site of sepsis, isolate the organism, and treat it with appropriate antibiotics promptly in critically ill T2DM patients to improve the outcomes of these patients.

## Introduction

Type 2 diabetes mellitus (T2DM) is a complex clinical syndrome that causes hyperglycemia due to decreased insulin secretion and insulin sensitivity in the body tissues [1]. Out of its numerous complications, the development of infections is one complication that has adverse effects on the mortality and morbidity of T2DM patients. Various metabolic derangements, like activation of various glycation end products, production of various free radicles, etc., hamper the immune system of patients with T2DM, hence making them extremely susceptible to contracting various infections [2]. T2DM severely depresses leucocyte activity, suppresses the antioxidant system, compromises the inherent immune system, and causes micro- and macroangiopathies, neuropathies, and several other complications [3,4].

Various complications of T2DM, like diabetic ketoacidosis, hyperglycemic hyperosmolar syndrome, and hypoglycemia, commonly occur with infections and often worsen the patient’s condition, leading to ICU admission for close monitoring [5]. Sepsis is one of the most common reasons for the ICU admission of T2DM patients. In the United States, approximately 1.7 million patients are admitted to the ICU annually due to sepsis [6]. Multiple factors predispose T2DM patients to severe sepsis. As discussed before, T2DM patients have an inherently compromised immune system. Patients in the ICU undergo various invasive procedures like endotracheal intubation, central line insertion, Foley catheterization, etc., which act as a portal of entry for several pathogens. Also, the presence of hyperglycemia in critically ill patients serves as an important risk factor for the development of sepsis [7].

Gram-negative bacteria (GNB) bacteria commonly elicit their pathogenicity in three phases. First, the bacteria invade the bloodstream via various mechanisms, such as capsule production or the secretion of endotoxins. The invaded bacteria then disseminate into the bloodstream due to several substances, such as adhesins and exotoxins, which are usually species-specific. It can also occur by activating various pathways in the epithelial cells, such as hypoxia-inducible factor 1-alpha. These bacteria survive and propagate in the body via metabolic flexibility and capsule production to protect the immune system. Sepsis caused by GNB is known to be more dangerous as it has the potential to elicit a stronger inflammatory response than gram-positive bacteria [8].

Studies have shown that the presence of T2DM in critically ill patients with sepsis leads to a higher incidence of the development of conditions like multi-organ dysfunction syndrome, which can lead to severe renal dysfunction, acute respiratory distress syndrome, or cardiac dysfunction [9].

The current study aimed to study the clinical and microbiological profile of T2DM patients with gram-negative sepsis admitted to the medical ICU (MICU). The inference of the study aids in characterizing the spectrum of gram-negative organisms encountered in critically ill diabetic patients with sepsis and analyzing their clinical outcomes, which would assist in better management of sepsis and improve clinical outcomes in critically ill diabetic patients.

## Materials and methods

This cross-sectional observational study was conducted on 100 patients diagnosed with sepsis who were admitted to the MICU at Dr. D. Y. Patil Medical College, Hospital & Research Centre, Pune, India, between December 2023 and May 2024, covering a period of six months. The study commenced after approval from the institutional ethics and scientific committee (approval number IESC/PGS/2022/16) and after obtaining fully informed written consent from the study participants.

Patients were divided into two groups: 50 T2DM patients and 50 nondiabetic controls. The inclusion criteria for the T2DM group were as follows: age ≥18 years, admission to the MICU with evidence suggestive of sepsis (as defined below) and confirmation of sepsis due to GNB, and a diagnosis of T2DM either on oral hypoglycemic drugs and/or insulin or meeting one of the following criteria upon admission to the MICU: fasting blood sugar level ≥126 mg/dl, random blood sugar ≥200 mg/dl, or hemoglobin A1c (HbA1c) ≥6.5%.

The inclusion criteria for controls were as follows: age ≥18 years, admission to the MICU with evidence suggestive of sepsis (as defined below), confirmation of sepsis due to GNB, and not meeting the criteria for T2DM outlined above. Sepsis was defined as a sequential [Sepsis-related] Organ Failure Assessment (SOFA) score of more than 2, along with any two of the following criteria: (1) temperature >38 °C or <36 °C; (2) heart rate >90/min; (3) respiratory rate >20/min or PaCO2 <32 mm Hg (4.3 kPa); and (4) white blood cell count >12,000/mm³ or <4,000/mm³ or >10% immature bands.

Patients under 18 years of age, pregnant patients, those with a recent history of organ transplants, and patients with HIV infection were excluded from the study.

The following investigations were conducted for all study subjects: complete hemogram (including hemoglobin, total leukocyte count, and platelet count), serum creatinine and urea levels, liver function tests (including total bilirubin, conjugated bilirubin, unconjugated bilirubin, aspartate transaminase, alanine transaminase, and international normalized ratio), serum electrolytes (sodium and potassium), serum procalcitonin, erythrocyte sedimentation rate (ESR), CRP, fasting and post-prandial blood sugar levels, and HbA1c estimation.

On admission, depending on the suspected site of infection, cultures were collected under full aseptic precautions from the following sites: peripheral/central venous samples, tracheal secretions, urine samples, ascitic fluid, cerebrospinal fluid, and pus from skin and soft tissue infections. The blood culture samples were collected in BACT-FX sterile containers and processed in the BACT-FX machine. The urine samples were collected in sterile containers and processed using cysteine-lactose-electrolyte-deficient agar. The pus samples were collected from the infected sites using sterile swabs and processed using blood agar and MacConkey agar. The ascitic and cerebrospinal fluids were collected in sterile containers and processed using blood agar and MacConkey agar. The bacteria from various specimens were identified based on colony morphology and biochemical reactivity.

Categorical variables were expressed in terms of frequency and percentages, and continuous variables were expressed as mean and SD (mean ± SD). The association between categorical variables was studied using the chi-square test. Student’s t-test was used to compare the mean of continuous variables between the cases and the control group. All the applied tests were two-tailed with a CI of 95%, and a p-value of less than 0.05 was considered statistically significant.

## Results

This study included 50 T2DM patients and 50 nondiabetic controls (who were admitted to the ICU and diagnosed with sepsis) to assess the clinical and microbiological profile of gram-negative infections. The mean age of the T2DM patients was 60.52 ± 12.88 years. In this study, among 50 critically ill T2DM cases with GNB sepsis, there were 28 males and 22 females, with a male-to-female ratio of 1.1:1. Most of the study subjects with T2DM were recently diagnosed (n = 35), and most of the patients were receiving oral hypoglycemic agents (n = 23). The most commonly associated comorbidity with T2DM in the study subjects was hypertension (n = 17), followed by chronic kidney disease (n = 8).

Respiratory system (n = 15) and central nervous system (n = 11) disorders like pneumonia, respiratory failure, stroke, meningitis, and several others were the common causes of ICU admission in the study. The average duration of ICU stay in critically ill T2DM cases was 13.34 ± 5.65 days, and the average duration of stay in nondiabetic controls was 12.3 ± 8.16 days, but this was statistically insignificant (p > 0.05). The median duration of the ICU stay was 13, with an IQR of 7. Around 30 (60%) critically ill T2DM cases had an ICU stay of more than 10 days. More than half of T2DM critically ill patients with sepsis due to GNB required invasive mechanical ventilation during their course of ICU stay (n = 28).

Table 1 shows the lab parameters of the critically ill subjects with GNB sepsis. It was seen that all lab parameters were comparable between T2DM cases and nondiabetic controls (p > 0.05) except for HbA1c, fasting blood glucose levels, and postprandial blood glucose levels, which were significantly higher in the T2DM group (p < 0.05). In this study, we found that inflammatory markers like ESR, CRP, serum lactate, and serum procalcitonin were elevated above normal limits among all the study subjects.

**Table 1:** Laboratory parameters in critically ill study subjects with GNB sepsis ALT, alanine transaminase; AST, aspartate transaminase; ESR, erythrocyte sedimentation rate; GNB, gram-negative bacteria; HbA1c, hemoglobin A1c; INR, international normalized ratio; NS, not significant; S, significant; T2DM, type 2 diabetes mellitus

Parameters (normal values)	Mean values in T2DM patients (± SD)	Mean values in nondiabetic controls (± SD)	p-value (Student’s t-test)
Hemoglobin (13.8-17.2 g/dl)	11.24 ± 2.57	10.97 ± 2.42	0.58 (NS)
Total leukocyte count (4,000-11,000 cells/µl)	11,210 ± 5,369.73	11,512 ± 5,280	0.78 (NS)
Platelet count (× 10^5^/µl) (1.5-4.5 × 10^5^/µl)	2.13 ± 1.50	2.45 ± 1.93	0.62 (NS)
CRP (<3 mg/L)	61.93 ± 53.93	67.16 ± 67.76	0.64 (NS)
ESR (<15 mm/hr)	39.46 ± 33.62	46.3 ± 38.75	0.34 (NS)
Serum procalcitonin (<0.1 ng/ml)	2.48 ± 3.47	3.93 ± 10.9	0.28 (NS)
Serum urea (7-20 mg/dl)	54.64 ± 65.88	48.08 ± 49.45	0.57 (NS)
Serum creatinine (0.74-1.35 mg/dl)	1.99 ± 2.06	1.51 ± 1.36	0.17 (NS)
Total bilirubin (0.1-1.2 mg/dl)	1.09 ± 1.01	1.74 ± 3.45	0.20 (NS)
AST (10-40 U/l)	116 ± 386.97	82.96 ± 167	0.57 (NS)
ALT (7-56 U/l)	163 ± 654.39	103.6 ± 216.17	0.53 (NS)
INR (0.8-1.2)	1.26 ± 0.43	1.35 ± 0.35	0.30 (NS)
Serum sodium (135-145 mmol/l)	133.64 ± 6.53	132 ± 7.56	0.28 (NS)
Serum potassium (3.5-5.0 mmol/l)	4.23 ± 0.95	4.07 ± 0.72	0.32 (NS)
Serum lactate levels (0.5-2.2 mmol/L)	2.02 ± 0.76	2.09 ± 0.75	0.63 (NS)
Fasting blood sugar (70-100 mg/dl)	204 ± 68	102 ± 17.54	<0.00001 (S)
Post prandial blood sugar (<140 mg/dl)	223 ± 77.6	106.82 ± 13.71	<0.00001 (S)
HbA1c (4-5.6%)	8.28 ± 1.68	4.81 ± 0.75	<0.00001 (S)

As shown in Table 2, in critically ill T2DM patients, bloodstream infections (n = 21) were more common, followed by bronchopneumonia (n = 16) and urinary tract infections (n = 10). Other sites of infection were the peritoneum (n = 1), skin (n = 1), and meninges (n = 1). In the case of nondiabetic controls, urinary tract infections were more common (n = 20) in a statistically significant manner (p < 0.05).

**Table 2:** Distribution of critically ill study participants with GNB sepsis according to the site of infection GNB, gram-negative bacteria; NS, not significant; S, significant; T2DM, type 2 diabetes mellitus

Site of infection	T2DM patients, n = 50 (%)	Nondiabetic controls, n = 50 (%)	p-value (chi-square test)
Bloodstream infection	21 (42)	12 (24)	0.055 (NS)
Pneumonia	16 (32)	16 (32)	1 (NS)
Urinary tract infection	10 (20)	20 (40)	0.290 (S)
Peritonitis	1 (2)	1 (2)	1 (NS)
Meningitis	1 (2)	1 (2)	1 (NS)
Skin and soft tissue infection	1 (2)	0 (0)	1 (NS)

Table 3 shows different GNBs isolated from the study subjects from different culture sites. Overall, *Escherichia coli *was the most common GNB organism isolated from the cases and nondiabetic controls. The most common isolates in critically ill T2DM cases with sepsis were *E. coli *(n = 15), *Klebsiella pneumoniae *(n = 15), and *Pseudomonas *spp. (n = 13). The most common GNB isolated in blood cultures of critically ill T2DM patients was *Acinetobacter *spp. (n = 6). The common organisms causing bronchopneumonia in critically ill T2DM cases were *Pseudomonas *spp. (n = 7), followed by *K. pneumoniae (*n = 4) and *Acinetobacter *spp. (n = 4). Most urinary tract infections in critically ill T2DM cases were caused by *E. coli *(n = 9).

**Table 3:** Pathogens responsible for sepsis in the critical study subjects with GNB sepsis GNB, gram-negative bacteria; T2DM, type 2 diabetes mellitus

Organisms isolated		Blood culture, n (%)	Tracheal aspirate culture, n (%)	Urine culture, n (%)	Other cultures, n (%)	Total n (%)
*Escherichia coli*	T2DM	5 (9.4)	1 (1.8)	9 (16.9)	0 (0)	15 (28.1)
	Non-T2DM	2 (4)	3 (6)	10 (20)	0 (0)	15 (30)
*Klebsiella pneumoniae*	T2DM	5 (9.4)	4 (7.5)	4 (7.5)	2 (3.7)	15 (28.1)
	Non-T2DM	4 (8)	4 (8)	5 (10)	2 (4)	15 (30)
*Pseudomonas*spp.	T2DM	5 (9.4)	7 (13.2)	0 (0)	1 (1.8)	13 (24.4)
	Non-T2DM	5 (10)	3 (6)	5 (10)	0 (0)	13 (26)
*Acinetobacter*spp.	T2DM	6 (11.3)	4 (7.5)	0 (0)	0 (0)	10 (18.8)
	Non-T2DM	1 (2)	6 (12)	0 (0)	0 (0)	7 (14)

Among the critically ill T2DM patients, 26 of the patients survived the duration of the ICU stay, whereas 24 patients succumbed to the illness. On the other hand, in the case of nondiabetic controls, 13 patients died and 37 survived. The death rate was significantly higher in T2DM patients with sepsis as compared to nondiabetic patients (chi-square test p-value = 0.022). *K. pneumoniae *and *Pseudomonas *spp. were the common organisms isolated in critically ill T2DM patients who succumbed to the illness.

## Discussion

The global burden of T2DM is on the rise. Currently, India houses almost 74.9 million T2DM patients, and this number is projected to increase to 124.9 million by 2045 [10]. With the increasing prevalence of T2DM, the prevalence of various complications related to T2DM like micro or macrovascular angiopathy, diabetic nephropathy, diabetic neuropathy, and sepsis is increasing too. Sepsis is defined as a systemic disorder caused by microbial invasion of normally sterile body organs [11]. Sepsis in T2DM patients is caused by many factors, such as an impaired immune system, a disrupted integumentary system, autonomic disturbances, and impaired microvascular circulation [12]. The risk of developing sepsis and other complications in T2DM patients depends on various factors such as age, BMI, poor glycemic control, etc. Age plays an important role in determining the chronicity of T2DM and has a direct influence on the complications associated with T2DM. Higher age and a later onset of T2DM pose a significant risk to the development of T2DM. In our study, the average age of the patients with T2DM was 60.52 ± 12.88 years. There are conflicting reports on the association between gender and the development of life-threatening sepsis in T2DM patients.

Another important risk factor for the development of life-threatening sepsis in T2DM patients is poor glycemic control. Studies have shown that there is a direct correlation between poorly controlled T2DM and the development of life-threatening infections and sepsis [13]. In our study too, almost all the admitted T2DM patients had poor glycemic control, with an average HbA1c of 8.28 ± 1.68. It is important to keep T2DM under check to reduce the chances of contracting life-threatening infections and to reduce the morbidity associated with them [14].

There are multiple reasons for T2DM patients to get admitted to the ICU. In the current study, respiratory illnesses like bronchopneumonia and respiratory failure were the most common indications for ICU admission. A study has shown that the presence of T2DM is a major risk factor for developing respiratory infections that require ICU admission [15]. Studies have shown that T2DM patients have a longer duration of ICU stay due to several reasons, such as the presence of more serious infections due to an impaired immune system, the presence of multiple comorbidities, and an increased risk of multiorgan dysfunction [16]. In the current study, 30 (60%) critically ill T2DM cases had an ICU stay for more than 10 days. In the current study, the majority of the GNB in T2DM patients was isolated from the blood cultures (n = 21), followed by tracheal aspirate cultures (n = 16). In a study by van Vught et al., it was observed that bloodstream infections and bronchopneumonia were more commonly encountered in critically ill patients [17].

The prevalence of gram-negative infections is increasing by the day [18]. Sepsis due to gram-negative infections is dangerous, especially in diabetics, as GNB is known to elicit a stronger immune response and show a wide spectrum of antibiotic resistance. In the current study, the most common GNB isolates in T2DM cases were *E. coli *(n = 15), *K. pneumoniae *(n = 15), and *Pseudomonas *spp. (n = 13). In a study by Benavent et al., commonly isolated gram-negative organisms from T2DM patients included *E. coli *and *Pseudomonas *spp. [19]. Critically ill T2DM patients with sepsis are prone to developing various life-threatening complications such as acute respiratory distress syndromes, multi-organ dysfunction syndromes, and acute kidney injury, which dramatically affect the outcome of the patients [20,21]. In our study, more than 50% of the critically ill T2DM patients with sepsis required mechanical ventilation (n = 28). Most of the patients also had deranged renal and liver function tests. This reflects the prevalence of the above-listed complications in our study. It is important to identify and treat these complications promptly to increase the odds of patient survival [22].

The most common organism isolated in T2DM patients who succumbed to the illness were *Pseudomonas *spp. and *K. pneumoniae*. Gram-negative organisms like *K. pneumoniae *and *Pseudomonas *spp. have high virulence and are associated with more mortality due to several factors such as high antibiotic resistance patterns, various pathophysiologic mechanisms such as polysaccharide capsules, dangerous exotoxins secreted by the bacilli, and the formation of protective biofilms [23]. As shown in a study by Bassetti et al., the presence of antimicrobial resistance significantly increases in-hospital patient mortality [24].

When the outcomes of the patients in this study were assessed, the frequency of death was significantly higher in critically ill T2DM patients with sepsis as compared to nondiabetic controls. These findings were in line with another study, which suggested that mortality in critically ill T2DM patients was significantly higher [25].

Although several studies exist that have analyzed various infections like diabetic soft tissue infections and bloodstream infections in T2DM patients separately, there is a dearth of studies that compare various sites of gram-negative infections in critically ill T2DM patients in a single, comprehensive study. With the ever-increasing prevalence of T2DM, conducting such studies on a larger scale is a need of the hour.

Limitations of the study

The limitations of our study include its single-center design, which may affect the generalizability of the findings to other hospital settings. Additionally, as a hospital-based study, it is susceptible to selection bias. The small sample size and short study duration also limit the scope and applicability of our conclusions. While univariate analyses were conducted on certain variables, a more comprehensive approach would involve performing a propensity score-matched analysis based on the APACHE II score, Charlson Comorbidity Index, and SOFA score. Furthermore, the resistance profiles of individual organisms were not assessed, which restricts the conclusions and broader applicability of the study.

## Conclusions

This study highlights the significant burden of gram-negative sepsis among patients with T2DM and its impact on their morbidity. The increasing prevalence of T2DM and its complications, including sepsis, underscores the need for a detailed exploration of this public health issue. T2DM patients are particularly susceptible to various invasive gram-negative infections that often result in ICU admissions. Common infections caused by gram-negative bacteria in these critically ill patients include bloodstream infections, bronchopneumonias, and urinary tract infections. *E. coli*, *K. pneumoniae*, and *Pseudomonas *spp. are frequently isolated gram-negative pathogens in this cohort. Gram-negative infections are known for inducing a potent inflammatory response and exhibiting widespread resistance to antimicrobial agents, factors that contribute to the high mortality associated with such infections, especially in T2DM patients. Therefore, prompt isolation, identification, and aggressive treatment of these organisms are crucial for improving patient outcomes and reducing both mortality and morbidity. A thorough analysis of the microbiological profile in these patients can assist physicians in managing sepsis more effectively in critically ill T2DM individuals.
